# Vitamin D and Depressive Symptoms in Adults with Multiple Sclerosis: A Scoping Review

**DOI:** 10.3390/ijerph19010199

**Published:** 2021-12-25

**Authors:** Carmen Concerto, Alessandro Rodolico, Alessia Ciancio, Christian Messina, Antimo Natale, Ludovico Mineo, Fortunato Battaglia, Eugenio Aguglia

**Affiliations:** 1 Psychiatry Unit, Department of Clinical and Experimental Medicine, University of Catania, 95123 Catania, Italy; alessandro.rodolico@me.com (A.R.); alessia.ciancio@gmail.com (A.C.); antimo.natale@yahoo.it (A.N.); ludwig.mineo@gmail.com (L.M.); eugenio.aguglia@unict.it (E.A.); 2 MS Center, Department “G.F. Ingrassia”, University of Catania, Via Santa Sofia 78, 95123 Catania, Italy; chry.messina@gmail.com; 3 Department of Medical Sciences, Neurology and Psychiatry, Hackensack Meridian School of Medicine, Nutley, NJ 07110, USA; fortunato.battaglia@hmhn.org

**Keywords:** depression, Multiple Sclerosis, vitamin D

## Abstract

Background. Vitamin D deficiency has been correlated with Multiple Sclerosis (MS) risk and disease activity. There is some controversy as to whether vitamin D could have an impact on depressive symptoms in people with MS (pwMS). The aim of this scoping review was to evaluate the association between vitamin D status and depressive symptoms in pwMS. Methods. We searched databases to include studies published up to March 2021 to provide an overview of the available evidence on the correlation between vitamin D status and depressive symptoms in pwMS. The eligibility criteria were as follows: studies evaluating the use of vitamin D measurement on depressive symptoms in patients suffering from MS, including randomized and non-randomized studies; studies written in English; and studies exploring an adult population over the age of 18. Results. Eleven studies met our inclusion criteria: two of them were abstracts only; the majority were cross-sectional studies; two were prospective longitudinal studies; one was a retrospective cohort study; and one was a randomized placebo-controlled trial (RCT). Of the eleven studies selected, seven showed a potential correlation between low vitamin D levels and depressive symptoms. Conclusion. Future RCT studies should include patients with greater severity of depressive symptoms and should consider confounding factors such as sun exposure and seasonal variation of vitamin D.

## 1. Introduction

Multiple Sclerosis (MS) is a neurodegenerative disease of the central nervous system (CNS), characterized by demyelination and axonal loss. Evidence from several sources indicates that it is associated with a complex interplay of genetic, immunologic, and environmental factors [1]. According to the most recent studies, about 2.8 million people worldwide have received a diagnosis of MS. MS typically affects young and middle-aged adults with a strong impact on general life functioning [2]. Late-onset MS (LOMS) may occur, albeit even if less frequently, with an estimated prevalence of about 10–20% of MS cases [3]. MS is clinically characterized by extreme inter and intra-variability in terms of clinical course with different clinical manifestations such as changes in sensation, vision, mobility, balance, and cognition. Based on the initial disease course, MS is classified as either relapsing–remitting MS (RRMS) or primary progressive MS (PPMS) [4,5]. The most frequent clinical course is RRMS, which accounts for approximately 80–85% of the initial diagnoses and is characterized by relapses followed by periods of remission [5]. People with MS (pwMS) may experience numerous symptoms, including spasticity, fatigue, cognitive dysfunction, depression, bladder dysfunction, bowel dysfunction, sexual dysfunction, and pain [6,7,8,9]. Indeed, MS has a multidimensional impact on personal life with symptoms that imply a significant loss of autonomy and may increase the risk of psychiatric illness [10]. Depression is one of the most common psychiatric illnesses among pwMS, with a lifetime prevalence estimated to be around 50% [11,12]. The symptoms may include loss of interest in daily activities, sadness, feelings of helplessness and hopelessness, concentration problems, sleep changes, and loss of energy [13,14]. Moreover, MS management may require lifelong pharmacological interventions [15,16] with different safety concerns due to the lifelong therapy [17,18]. The worsening of depressive symptoms can reduce the compliance of patients who take disease-modifying treatments (DMTs) [19]. Psychological and pharmacological approaches have been suggested for the treatment of depression in pwMS with evidence showing a widespread use of antidepressant medications and a clear need for further investigation [12].

Recently, attention has been given to non-pharmacological treatment for the maagement of depression in MS, focusing on Complementary and Alternative Medications (CAMs) [20,21,22]. CAM is a heterogeneous group of treatments that includes herbal therapies and dietary supplements, previously studied in healthy individuals [23,24,25] and in pwMS [20,21,25]. Among the CAM, attention has been given to vitamin D, a lipid-soluble vitamin mostly produced from exposure of the skin to sunlight; it is also acquired via dietary intake. The metabolite of vitamin D that is abundant in the circulation is 25-hydroxyvitamin D (25(OH)D); it is also considered to better describe vitamin D status [26].Vitamin D deficiency is currently considered to be one of the environmental risk factors for the pathogenesis and progression of MS [27]. Vitamin D presents an immune-modulatory effect able to induce an increase in anti-inflammatory cytokines and a decrease in pro-inflammatory cytokines [28]. Previous studies have shown that vitamin D might act in MS by stimulating interleukin (IL)-10 cytokine levels and by reducing IL-17 cytokine levels and the B-cell immunoreactivity [27]. Indeed, it has a favorable impact on the inflammatory pathways of MS and in pwMS; moreover, high circulating levels of vitamin D have been associated with lower risk of disease [20,29]. 

Vitamin D deficiency has been considered a risk factor for the presence and severity of depressive symptoms in both depressed patients and those with pwMS [30,31,32,33]. In depressed patients, increased levels of pro-inflammatory cytokines level-tumor necrosis factor alpha (TNFα), interleukin (IL)-1 and IL-6– have been reported in the CNS and peripheral circulation [12,34]. Previous studies have shown a beneficial effect of vitamin D supplementation on depressive symptoms, supporting the idea that a poor vitamin D status may contribute to depression [35,36]. The association of depressive symptoms with vitamin D supplementation or serum vitamin D levels is less established in pwMS [35,37]. Hence, considering the context of the chronic inflammation in MS, vitamin D status and its supplementation have been studied to evaluate the effect on depressive symptoms. The objective of this scoping review was to provide an overview of the available evidence on the correlation between vitamin D status and depressive symptoms in pwMS. In particular, we wanted to answer the following question: Is there an association between vitamin D status and depressive symptoms in pwMS?

## 2. Methods

### 2.1. Search Strategy

In performing this scoping review, we followed the PRISMA-ScR (Preferred Reporting Items for Systematic Reviews and Meta-Analyses Extension for Scoping Reviews) guidelines [38]. A preliminary search for existing scoping reviews (and ideally systematic reviews too) on the topic was conducted. The review included five steps: (1) defining the research question; (2) identifying relevant studies; (3) selecting studies; (4) charting the data; and (5) collecting, summarizing, and reporting the results. 

### 2.2. Search and Studies Selection

On 23 March 2021, we conducted systematic searches in MEDLINE (accessed through PubMed), EMBASE, by using the following search string: (“Vitamin D” OR cholecalciferol OR colecalciferol OR calcitriol OR 1,25-dihydroxycholecalciferol) AND “multiple sclerosis” AND (depress* OR mood*). The eligibility criteria were as follows: (1) studies evaluating the use of vitamin D measurement on depressive symptoms in patients suffering from MS, including randomized and non-randomized studies; (2) studies written in English; (3) studies exploring an adult population, over the age of 18. We excluded studies referring to (1) correlation between general depression and vitamin D, with no mention of MS; (2) correlation between general MS symptoms and vitamin D, with no mention of depression. Two independent reviewers (AC and AN) initially screened the titles and abstracts, and then selected the studies to be included after checking the full text. In case of disagreement, the final decision was made with the help of a third reviewer (either CC or AR). The interrater agreement in the title/abstract and full text selection phases was fair (kappa for agreement 0.83 and 0.86, respectively). Consequently, the relevant data were extracted in a predefined form. The following characteristics were collected: first author and year of publication, country where the study was conducted, study design, study aim, study duration, study population characteristics, method used for vitamin D level measurement, main study results, and cofounding factors (i.e., age, sex, degree of disability, fatigue, sun exposure). After double-checking the extracted data, the resulting forms were merged in one comprehensive table, grouped by study design.

#### Quality Assessment

The scoping review considered observational (cross-sectional and cohort) studies and interventional studies. We adopted the appropriate quality assessment tool depending on the study design. In particular, we used the original version of the Newcastle-Ottawa scale for cohort studies [39]. an adapted version of the Newcastle-Ottawa scale developed by Herzog and colleagues [40] for cross-sectional studies. We adopted the National Institutes of Health (NIH) quality assessment tool for before–after (Pre–Post) study with no control group, for arm interventional studies [41]. Finally, we assessed the randomized controlled trials quality by using the Cochrane Risk of Bias 2.0 [42].

## 3. Results

**Figure 1:**
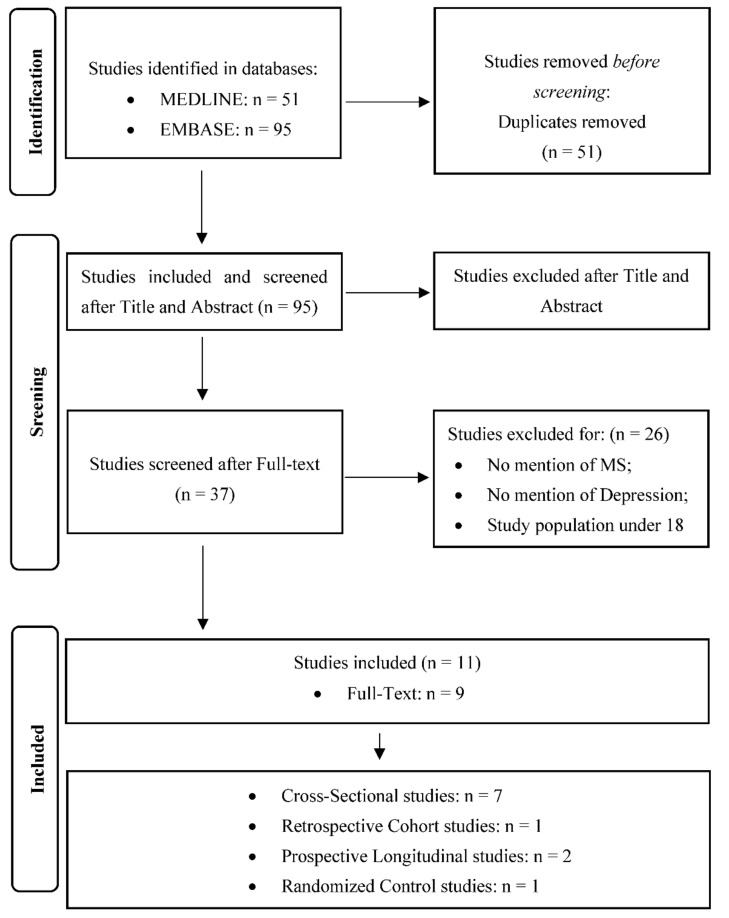
PRISMA flowchart.

The PRISMA flow chart is shown in Figure 1. A total of 95 potentially relevant studies were identified. 

After the title screening, 58 were excluded, leaving 37 studies. Of the 37 studies that were screened for eligibility, only 11 were considered eligible and included. Among them, nine were full text articles, two were abstracts, seven were cross-sectional studies [26,43,44,45,46,47,48] one was a retrospective cohort study [49] and two were prospective longitudinal studies [50,51]. Only one study was a RCT [52]. The included studies were conducted in various countries (Iran, Portugal, Argentina, Romania, Jordan, Australia, the Netherlands, and Saudi Arabia). The earliest publication date was 2012 and the most recent was 2021. Most of the studies assessed a population of patients with RRMS. Some studies explored if there was a correlation between vitamin D serum level -defined as vitamin D status-and depressive symptoms, meanwhile other studies reported the effect of vitamin D supplementation on depression in MS. Table 1 presents a detailed summary of the core characteristics of the included studies.

### 3.1. What Is the Association between Circulating Levels of Vitamin D and Symptoms of Depression?

In our scoping-review we found that four studies suggested a potential beneficial association between vitamin D circulating levels of vitamin D and depressive symptoms in pwMS. For instance, Ashtari et al. [43] found that low serum vitamin D level was inversely associated with depression scores. Similar results were reported by the studies of Knippenber et al. [48] and Silva et al. [45]. El Salem 2021 et al. [26] found a significant correlation between serum vitamin D level and scores of depression scales in males but not in females. Other studies [44,46] did not find a significant association between vitamin D levels and depression. 

### 3.2. What Is the Association between Vitamin D Supplementation or Sun Exposure on Depression Symptoms?

Freitas et al. [44] reported that sun exposure might have had an impact on depressive symptoms. In particular, they suggested that people with more depressive symptoms would refrain from outdoor activities, and this could account for the apparent relationship between sun exposure and depression. Knippenberg et al. [51] found that vitamin D levels were inversely associated with depression scores, but they related their results to sun exposure and not to vitamin D status.

Regarding the effect of vitamin D supplementation on depressive symptoms in pwMS, Taylor 2014 et al. [47] and Taylor 2018 [49] found that vitamin D supplementation reduced depression risk for pwMS. These findings were confirmed by Kotb et al. study [50]. The authors reported that lower vitamin D levels were associated with higher depressive scores and suggested that vitamin D replacement could have improved depressive symptoms in patients with RRMS. In the randomized controlled study by Rolf et al. [52], vitamin D supplementation did not affect depressive symptoms in the study group compared to the placebo group. 

### 3.3. Study Design Cross-Sectional Studies

Ashtari et al. [43] assessed the relationship between vitamin D status, fatigue, and depressive symptoms. They referred to serum vitamin D as normal or low and they found that a low serum vitamin D level was inversely associated with depression scores. Their findings in this regard were similar to the studies of Knippenber et al. [48] and Silva et al. [45]. In their study, Knippenberg et al. [48] observed that vitamin D status was negatively correlated with depressive symptoms. However, after controlling for age, Expanded Disability Status Score (EDSS), and fatigue the statistical significance was lost. Silva et al. [45] evaluated the relationship between serum vitamin D levels, cognitive impairment, depression and fatigue. The authors found potential correlation between low vitamin D levels and depression, fatigue, and cognitive impairment. Freitas et al. [44] reported low vitamin D levels in 42.6% of MS patients, no correlation between vitamin D levels and depression was found. Tiu et al. [46] investigated the relationship between vitamin D status, depression and self-reported impact of disease. They did not find any significant association between low vitamin D levels and depression. El Salem 2021 et al. [26] observed a significant correlation between serum vitamin D levels and scores of depression scales in males but not in females. Taylor 2014 et al. [47] studied the association between modifiable lifestyle factors and risk of depression in MS patients. They found that vitamin D supplementation reduced depression risk for people with MS. In their follow-up study, the authors investigated whether modifiable lifestyle factors were associated with depression over 2.5 years of follow-up. Their results showed that vitamin D supplementation was associated with a reduction in the risk of developing depression [49].

#### Longitudinal Studies

The prospective observational study by Kotb et al. [50] explored whether depression in MS was related to vitamin D deficiency and whether the replacement of vitamin D would have improved depressive symptoms in this group of patients. Their results showed that lower vitamin D levels were associated with higher depressive scores and that vitamin D replacement could improve depressive symptoms in patients with RRMS. The prospective longitudinal study by Knippenberg et al. [51] examined the associations between sun exposure, serum vitamin D, depression, anxiety, fatigue, and cognition in MS patients. The authors found that vitamin D levels were inversely associated with depression scores, but that this was not significant after adjustment for patient-reported sun exposure. The randomized controlled study by Rolf et al. [52] explored the effect of high-dose vitamin D supplementation on depressive symptoms and on pro- and anti-inflammatory cytokine secretion by peripheral blood mononuclear cells (PBMC), PBMC and CD8+ T cells. Although a significant decrease in depressive symptoms was observed within the vitamin D supplementation arm, this reduction was not significantly different from the decrease seen in the placebo group. After 48 weeks of treatment with vitamin D supplementation, there was no reduction in the depressive symptoms or related pro- and anti-inflammatory cytokine balances secreted by stimulated leukocytes and CD8+ T cells.

## 4. Discussion

This study was carried out to provide an updated summary of previous evidence on the association between vitamin D status and depression in pwMS. Previous systematic reviews and meta-analysis on vitamin D and MS have focused on the impact of vitamin D on fatigue, disability status, and relapsing symptoms [30,53] evaluating the effects on depressive symptoms as a secondary outcome. To our knowledge, this is the first review that has investigated the relationship between vitamin D and depressive symptoms in pwMS. Among the studies correlating vitamin D serum level with depressive symptoms, Ashtari et al. [43], Knippenberg et al. [48], Silva et al. [45], and El Salem 2021 et al. [26], found that low serum circulating levels of vitamin D were inversely associated with depression scale scores. The reported effect sizes ranged between small and medium values. The other two correlational studies revealed a potential negative link between vitamin D levels and depression [44,46]

Studies on vitamin D supplements used as a treatment for depression in MS present conflicting results. Taylor et al. [47,49] showed that vitamin D supplementation was associated with a change in the risk for depression. However, their data may be limited by the study design. Indeed, they conducted an online survey with a heterogenous sample between the time points. Kotb et al. [50] found that vitamin D supplementation improved depressive symptoms in patients with RRMS. However, they reported data on a relatively small number of patients and the authors explained that the sample was too small to confirm the existence of such important association. The other studies investigating the role of vitamin D supplementation reported non-significant effect sizes. In particular, the only available RCT explored the effect of vitamin D supplementation on depressive symptoms and anti-inflammatory cytokine levels [52]. The data from this RCT study does not support the role of vitamin D in affecting depressive symptoms. Their inconclusive finding should be considered in light of the original aim of their project. The study participants presented a median Hospital Anxiety and Depression Scale (HADS) of 3 and 4 for active and control arms, respectively. Therefore, if a high evidence output might answer our scoping review question, their findings are not easily generalizable to patients with depression and MS in comorbidity [54].

Considering potential confounding factors, it appears that sun exposure, age, sex, degree of disability, and fatigue represent the most common factors, which could have influenced results of the studies included. However, Ashtari et al. [43] and Knippenberg et al. [51] reported a significant correlation between vitamin D status and depressive symptoms but not between vitamin D and fatigue. Moreover, Ashtari et al., did not consider the seasonal variation of vitamin D and they did not consider the other confounding factors [43]. Meanwhile, Knippenberg et al. found no significance after controlling for EDSS, age, and fatigue [48]. Regarding the disability status in MS patients, one recent study found that vitamin D supplementation had no effects on EDSS [55]. These findings are confirmed by a meta-analysis by Hanaei et al. [53]. In another study by Knippenberg et al., the authors reported conflicting results when they compared serum vitamin D levels as a continuous or categorical variable. They found a significant negative correlation with depressive symptoms only when vitamin D serum levels were higher than 80 nmol/L. Vitamin D deficiency was associated with depressive symptoms, but sun exposure had a moderating effect on them [51]. Moreover, in their study, Tiu et al. in their study found no significant associations between low vitamin D levels and depression. Additionally, vitamin D deficiency was associated with worse physical and psychological outcome measures [46]. Yet, El-Salem et al. [26] reported on the one hand a stronger negative correlation between serum vitamin D and depression scores in males, and on the other hand, they found that disability measures, such as EDSS, were correlated with females’ vitamin D levels in females only.

We do not know the exact mechanisms behind the association between vitamin D deficiency and depression, but there are several theories. According to Geng et al., it has been shown that a deficiency of vitamin D could affect the synthesis of serotonin, dopamine, and noradrenaline in the hippocampus, substantia nigra and prefrontal cortex [56]. Other authors have shown that vitamin D is involved in controlling the expression of those genes that are responsible for maintaining both Ca^2+^ and reactive oxygen species (ROS) homeostasis [57]. Moreover, vitamin D deficiency was linked to high levels of Ca^2+^ and ROS, which might affect neuronal cells and explain the link with depression. Indeed, an increase in the formation of ROS may exert a profound effect on neuronal function and has been observed in depression [32].

The mechanism by which vitamin D antidepressant therapeutic effect might be mediated remains uncertain. Both biological and psychological factors are thought to play a role in the etiology of depression in MS. A growing body of evidence highlights the role of a possible imbalance in pro- and anti-inflammatory cytokines in the development of depression in MS [58,59,60]. Moreover, the pro-inflammatory immunological changes suggested in depression may be linked to the disease activity, thereby worsening MS symptoms [61]. Preclinical literature supports the role of vitamin D on brain function. It has been demonstrated that vitamin D increases the brain concentration of brain-derived neurotrophic factor (BDNF) [62], which is a neurotrophic factor that modulates plasticity, mood, and the effects of antidepressants [63]. In addition, vitamin D modulates several brain areas, including the prefrontal cortex [64], which is an important brain area in the pathogenesis of depression and a target of newly non-pharmacological treatments [65,66]. Lastly, vitamin D is a strong regulator of brain morphology and neurogenesis [67,68]. Previous studies indicate that animal models of both depression and MS show abnormal hippocampal neurogenesis and neuronal morphology [69] and that morphological brain plasticity (neurogenesis, dendritic spine density, and dendritic complexity) is modulated by antidepressant treatments [70,71]. On the whole, preclinical results point to a brain plasticity modulating activity of vitamin D and support its role in the pathogenesis of depression in pwMS. Furthermore, regarding depressive symptoms in patients with MS and in depression in general, the data on Vitamin D still appear still unclear [58]. Indeed, the differences among the studies may be related in part to the small sample size, the seasonal variation of vitamin D and the different depressive symptoms scores at baseline. Moreover, the cross-sectional design studies we included may only establish an association but not causality. We should emphasize the fact that some studies did not perform all assessments on the same day and most of the studies did not examine confounding factors, such as sex differences. This is of pivotal importance as previous studies indicate sex difference in perceived stress [72], which was previously associated with depressive symptoms in MS [73]. This study presents several limitations: the search did not take into consideration grey literature or non-English studies due to lack of resources. In addition, we did not register a study protocol. The main reason was the exploratory nature of the present work. The strengths of the study are the novelty of the systematic approach in terms of search and data extraction, the evaluation of confounding factors, and the evidence gradient that underlines the need for further research.

## 5. Conclusions

The scoping review identified eleven studies that provided information on the role of vitamin D in affecting depressive symptoms in MS. Although some studies suggested a potential beneficial correlation between vitamin D and depression in pwMS, the results from cross-sectional and prospective studies are inconclusive. Future RCT studies on vitamin D and depressive symptoms in MS should include patients with actual depressive symptoms scores at baseline and should consider confounding factors, such as sun exposure and seasonal variation of vitamin D.

## Figures and Tables

**Table 1:** Included studies.

Author, Year (Country) Study Design (D) Recruitment Timing (RT ) Followup (FU) Study Population Characteristics	Vitamin D Measurements	Depression Measuremnts	Other Measured Variables	Study Aim(s) Results Cofounder Quality Assessment
Ashtari F. et al., 2013 (Iran) D: Cross-sectional study RT: March 2011 to September 2011 FU: NR Participants: 200 Mean Age (SD): 33.5 (8.4) Female/Male: 154/46 MS Diagnosis: McDonald’s criteria Clinical status: 94% of patients have a RR MS, relapse free for more than 8 weeks prior to the study selected.	Analyzed fluid: venous blood Analythical methodology: radioimmunoassay (RIA) method using Biosource kit Vit. D concentration (nmol/L): 76 (IQR: 35.5–100.7) Vit. D status: 51.5% normal vit. D level (>75 mmol/L), 49.5% low vit. D level (<75 mmol/L) Dietary vit. D intake: NA	Evaluation of depressive symptoms: Beck Depression Inventory for Primary Care (BDI-PC) Depressive symptoms severity: 4 (3–7) in the overall sample	Sun exposure: Not Measured Fatigue Diagnosis: Fatigue Severity Scale (FFS) Degree of disability: Expanded Disability Status Score (EDSS)	Aim: To investigate the relation between vitamin D status with fatigue and depressive symptoms Results: Vitamin D status was inversely associated with depressive symptoms. Effect size: correlation, β = −0.16, *p*-value = 0.02 Controlled cofounders: not considered Quality asessment (NOS—cross sectional studies adaptation):Selection (max 5): 3 - Representativenes: 1 - Sample size: 0 - Non-respondents: 0 - Exposure: 2 Comparability (max 2): 0 Outcome (max 3): 2 - Assesment: 1 - Statistical test: 1 Total score (max 10): 5
Knippenberg et al., 2011 (The Netherlands) D: Cross-sectional study RT: 2005–2007 FU: NR Participants: 59 Mean Age (SD): 44.2 (9.2) Female/Male: 16/43 MS Diagnosis: McDonald’s criteria Clinical status: Participants had not used corticosteroids for ≥ 4 weeks and were relapse free for ≥4 weeks prior to assessment.	Analyzed fluid: serum Analythical methodology: NA Vit. D concentration (nmol/L): 62.3 (SD 27.8) Vit. D status: 23% normal vit. D level (>75 mmol/L), 73% low vit. D level (<75 mmol/L) Dietary vit. D intake: NA	Evaluation of depressive symptoms: Hospital Anxiety and Depression Scale, depression subscale (HADS-D) Depressive symptoms severity: 6.2 (SD 4.4)	Sun exposure: Not Measured Fatigue Diagnosis: Multidimensional Fatigue Inventory (MFI) Degree of disability: Expanded Disability Status Score (EDSS)	Aim: To assess whether vitamin D status contributes to the presence of depressive symptoms and fatigue in MS. Results: Vitamin D status correlated negatively with depression. In a multiple regression model, vitamin D status was not a significant contributor to depression. Effect size: correlation, β = −0.33, *p*-value = 0.006 Controlled cofounders: age, EDSS and MFI scors Quality asessment (NOS—cross sectional studies adaptation): Selection (max 5): 2 - Representativenes: 0 - Sample size: 0 - Non-respondents: 0 - Exposure: 2 Comparability (max 2): 1 Outcome (max 3): 2 - Assesment: 1 - Statistical test: 1 Total score (max 10): 5
Silva et al., 2016 * (Argentina) D: Cross-sectional study RT: NA FU: NR Participants: 61 Mean Age (SD): 42.8 (12.4) Female/Male: 44/17 MS Diagnosis: NA Clinical Status: Relapsing Remitting MS (RRMS).	Analyzed fluid: serum Analythical methodology: chemiluminescence Vit. D mean (SD) (nmol/L): NA Vit. D status: 34% vit. D level (≤49.92 mmol/L), 66% low vit. D level (>49.92 mmol/L) Dietary vit. D intake: NA	Evaluation of depressive symptoms: Beck Depression Inventory II (BDI II). Depressive symptoms severity: NA	Sun exposure: Not Measured Neuropsychological status: Brief Repeatable Neuropsychological Battery (BNS-EM) Fatigue Diagnosis: Fatigue Severity Scale (FSS) Degree of disability: Expanded Disability Status Score (EDSS)	Aim: To evaluate the relationship between serum vitamin D levels and cognitive impairment, depression and fatigue Results: [An association between depression severity and vitamin D levels was observed (*p* < 0.01, X^2^ = 30.7). Effect size: odds ratio not reported Controlled cofounders: not considered Quality asessment (NOS—cross sectional studies adaptation): Selection (max 5): 2 - Representativenes: 0 - Sample size: 0 - Non-respondents: 0 - Exposure: 2 Comparability (max 2): 0 Outcome (max 3): 3 - Assesment: 2 - Statistical test: 1 Total score (max 10): 5
Freitas et al., 2017 (Portugal) D: Cross-sectional study RT: May 2016–January 2017 FU: NR Participants: 54 (78 study participants, 24 without vit. D data) Mean Age: NA Female/Male: NA MS Diagnosis: McDonald’s criteria Clinical Status: 55% RR MS.	Analyzed fluid: serum Analythical methodology: NA Vit. D mean (SD) (nmol/L): 78.7 (SD 48.9) Vit. D status: 27.8% vit. D level deficiency (≤49.92 mmol/L), 42.6% low vit. D level (49.92–72.4 mmol/L), 29.6% normal vit. D levels (>72.4 mmol/L) Dietary vit. D intake: 34.6% were taking vit. D continuing supplement	Evaluation of depressive symptoms: Hospital Anxiety and Depression questionnaire (HADS-D) Depressive symptoms severity: NA	Sun exposure: recall questionnaire assessing daily time in sun and skin exposure (face, limbs, body) for the previous week Fatigue Diagnosis: Modified Fatigue Impact Scale (MFIS) Daytime sleepiness: Epworth Sleepiness Scale (ESS) Degree of disability: Expanded Disability Status Score (EDSS)	Aim: To evaluate the prevalence and severity of fatigue and its relationship with other clinical variables. Results: No association between 25-OH-D levels and depression. Effect size: correlation not reported Controlled cofounders: not considered Quality asessment (NOS—cross sectional studies adaptation): Selection (max 5): 2 - Representativenes: 0 - Sample size: 0 - Non-respondents: 0 - Exposure: 2 Comparability (max 2): 0 Outcome (max 3): 1 - Assesment: 1 - Statistical test: 0 Total score (max 10): 3
Tiu et al., 2017 * (Romania) D: Cross-sectional Study RT: NA FU: NR Participants: 106 Mean Age (SD): 38.7 (10.1) Female/Male: 72/34 Clinical Status: patients treated with immunomodulatory drugs.	Analyzed fluid: serum Analythical methodology: chemiluminescence Vit. D mean (SD) (nmol/L): 57.9 (SD 29.5) Vit. D status: 81.1% vit. D level deficiency (≤74.9 mmol/L) Dietary vit. D intake: NA	Evaluation of depressive symptoms: Beck Depression Inventory for Primary Care (BDI-PC) Depressive symptoms severity: NA	Sun exposure: Not Measured	Aim: To investigate the relationship between vitamin D status, depression and self-reported impact of disease Results: no significant association between low vitamin D levels and depression Effect size: odds ratio not reported Controlled cofounders: not considered Quality asessment (NOS—cross sectional studies adaptation): Selection (max 5): - Representativenes: 0 - Sample size: 0 - Non-respondents:0 - Exposure: 2 Comparability (max 2): 0 Outcome (max 3): 3 - Assesment: 2 - Statistical test: 1 Total score (max 10): 5
El Salem et al., 2021 (Jordan) D: Cross-sectional study RT: October 2018–June 2019 FU: NR Participants: 88 Mean Age (SD): 36 (10.69) Female/Male: 64/24 MS Diagnosis: 2017 revised McDonald MS diagnostic Clinical Status: Being relapse-free for 30 days prior to participation.	Analyzed fluid: blood Analythical methodology: Enzyme-Linked Immunosorbent Assay (ELISA) Vit. D mean (SD) (nmol/L): 60.2 (SD 25.9) Vit. D status: 40.9% vit. D level deficiency (≤49.9 mmol/L), 27.3% low vit. D level (49.9–74.9 mmol/L), 31.8% normal vit. D levels (>74.9 mmol/L) Dietary vit. D intake: NA	Evaluation of depressive symptoms: Hospital Anxiety and Depression Scale (HADS-D); Beck Depression Inventory-II rating scale(BDI-II) Depressive symptoms severity: HADS-D: 8.45 (SD 5.28); BDI-II: 17.93 (SD 11.82)	Sun exposure: Not Measured Degree of disability: Expanded Disability Status Score (EDSS) Perceived level of motor disability: Patient-determined disease steps (PDDS)	Aim: To evaluate vitamin D levels and its correlation with validated depression scales. Results: Serum Vit. D levels significantly correlated with scores of depression scales regardless of sex. The HADS depression score significantly correlated with serum Vit. D levels. Similarly, a significant inverse association between BDI-II score and Vit. D level was noted. Effect size: Pearson correlation coefficient, r = −0.513, *p* < 0.001 (for HADS), r = −0.401, *p* < 0.001 (for BDI-II) Controlled cofounders: age, sex, BMI, duration of disease, type of MS, and EDSS Quality asessment (NOS—cross sectional studies adaptation): Selection (max 5): 2 - Representativenes: 0 - Sample size: 0 - Non-respondents: 0 - Exposure: 2 Comparability (max 2): 1 Outcome (max 3): 3 - Assesment: 2 - Statistical test: 1 Total score (max 10): 6
Taylor et al., 2014 (Australia) D: Cross-sectional study RT: NA FU: NR Participants: 2225 Median Age (IQR): 45 (38–53) Female/Male: 388/1813 MS Diagnosis: self-report Clinical Status: Being relapse-free for 30 days prior to participation, the majority (61.3%) had RR MS.	Analyzed fluid: not measured Analythical methodology: NA Vit. D mean (SD) (nmol/L): NA Vit. D status: NA Dietary vit. D intake: 30.1% no daily vit. D consumption, 17.4% 1–5000 IU daily vit. D consumption, >5000 IU vit. D consumption.	Evaluation of depressive symptoms: Patient Health Questionnaire depression module short version (PHQ-2) Depressive symptoms severity: NA	Sun exposure: Not Measured Fatigue Diagnosis: The Fatigue Severity Scale (FSS) Perceived level of disability: Patient-determined disease steps (PDDS) Habits Description: Diet Habits Questionnaire (DHQ) removed four items assessing salt use and alcohol intake; International Physical Activity Questionnaire (IPAQ) Comorbidities Assessment: Self-Administered Comorbidity Questionnaire (SCQ)	Aim: To examine depression and its association with modifiable lifestyle risk factors In the lifestyle factors: Vitamin D supplementation. Results: Taking any vitamin D supplement was associated with lower odds of screening positive for depression, but taking at least 5000 IU daily was associated with the greatest odds. Effect size: adjusted odds ratio 0.57 (0.43–0.77) for 1–5000 IU, 0.47 (0.32–0.70) for >5000 IU. Controlled cofounders: years since diagnosis, number of comorbidities, level of disability, clinically significant fatigue, age, gender, marital status and level of education. Quality asessment (NOS—cross sectional studies adaptation): Selection (max 5): 2 - Representativenes: 1 - Sample size: 1 - Non-respondents: 0 - Exposure: 0 Comparability (max 2): 1 Outcome (max 3): 2 - Assesment: 1 - Statistical test: 1 Total score (max 10): 5
Kotb et al., 2019 (Saudi Arabia) D: Prospective cohort study RT: 5 years (2013–2018) FU: All patients were regularly followed up every 2 months for 25-OH-D serum levels. EDSS scores and BDI scores. Participants: 35 Mean Age (SD): 27 (4). Female/Male: 19/16 MS: Diagnosis: McDonald’s criteria Clinical Status: Clinical Status: Being relapse-free for 30 days prior to participation, not receiving any corticosteroid therapy within four weeks prior to recruitment.	Analyzed fluid: serum Analythical methodology: NA Vit. D mean (SD) (nmol/L): baseline 23.4 (SD 9.8); endpoint 86.3 (SD 7.3) Vit. D status: NA Dietary vit. D intake: daily intake 1000 IU, for 3 months NB: patients with current MS treatment other than interferon, received high-dose vitamin D (daily intake 1000 IU) before inclusion to the study.	Evaluation of depressive symptoms: Beck Depression Inventory (BDI) Depressive symptoms severity: baseline 21.3 (SD 3.4); endpoint 16.8 (SD 2.9)	Sun exposure: Not Measured Degree of disability: Expanded Disability Status Score (EDSS)	Aim: evaluate the relation between vitamin D levels and depression scores, and the effect of vitamin D replacement on the depressive symptoms in patients with MS. Results: A significant negative correlation was observed between vitamin 25 (OH) D levels and Beck’s depression inventory scores at baseline (*p* < 0.001), eighth, tenth, and twelfth month (*p* = 0.001). Effect size: Pearson correlation coefficient r = −0.432 (*p*-value = 0.011) Controlled cofounders: EDSS Quality assessment (NIH quality assessment tool for before-after (Pre-Post) study with no control group): Was the study question or objective clearly stated? Yes Were eligibility/selection criteria for the study population prespecified and clearly described? Yes Were the participants in the study representative of those who would be eligible for the test/service/intervention in the general or clinical population of interest? Yes Were all eligible participants that met the prespecified entry criteria enrolled? Not reported Was the sample size sufficiently large to provide confidence in the findings? No Was the test/service/intervention clearly described and delivered consistently across the study population? Yes Were the outcome measures prespecified, clearly defined, valid, reliable, and assessed consistently across all study participants? Yes Were the people assessing the outcomes blinded to the participants’ exposures/interventions? No Was the loss to follow-up after baseline 20% or less? Were those lost to follow-up accounted for in the analysis? No Did the statistical methods examine changes in outcome measures from before to after the intervention? Were statistical tests done that provided p values for the pre-to-post changes? Yes Were outcome measures of interest taken multiple times before the intervention and multiple times after the intervention (i.e., did they use an interrupted time-series design)? Yes If the intervention was conducted at a group level (e.g., a whole hospital, a community, etc.) did the statistical analysis take into account the use of individual-level data to determine effects at the group level? Not aplicable Quality Rating: Fair
Taylor et al., 2018 (Australia) SD: Retrospective cohort study RT: NA FU: 2.5 years Participants: 1401 Mean Age (SD): 48.4 (10.5) Female/Male: 1150/241 Diagnosis MS: Clinical Status: Being relapse-free for 30 days prior to participation, the majority (59.3%) had RR MS.	Analyzed fluid: NA Analythical methodology: NA Vit. D mean (SD) (nmol/L): NA Vit. D status: NA Dietary vit. D intake: Vitamin D supplementation self-reported.	Evaluation of depressive symptoms: patient Health Questionnaire-2 (PHQ-2) at baseline, and Patient Health Questionnaire-9 (PHQ-9) at follow-up Depressive symptoms severity: NA	Sun exposure: Not Measured Fatigue Diagnosis: The Fatigue Severity Scale (FSS) Perceived level of disability: Patient-determined disease steps (PDDS) Habits Description: Diet Habits Questionnaire (DHQ) removed four items assessing salt use and alcohol intake; International Physical Activity Questionnaire (IPAQ) Severity of disease: Patient Determined Multiple Sclerosis Severity Score (P-MSSS)	Aim: Evaluating whether modifiable lifestyle factors were associated with screening positive for depression 2.5 years after our baseline study and the predictors of change in depression screen during follow-up. In the lifestyle factors: Vit D supplementation. Results: Vitamin D supplementation was associated with lower frequencies of depression risk by both PHQ-2 and PHQ-9. Effect size: adjusted odds ratio 0.70 (0.56–0.87) (*p*-value = 0.002) Controlled cofounders: age, P-MSSS, FSS, and use of antidepressant medication Quality assessment (NOS): Selection (max 4): 3 - Representativeness: 1 - Selection of the non exposed: 1 - Ascertainment of exposure: 1 - Outcome not present at start: 0 Comparability (max 2): 1 Outcome (max 3): 2 - Assesment: 0 - Follow-up lenght: 1 - Adequacy of follow up: 1 Total score (max 9): 6
Knippenberg et al., 2013 (The Netherlands) D: Prospective longitudinal study RT: (2002–2005) FU: 2.3 years Participants: 198 Mean Age (SD): 48.2 (11.4) Female/Male: 137/ 61 Diagnosis MS: McDonald’s criteria Clinical Status: Being relapse-free for 30 days prior to participation, 75.3% have RR MS.	Analyzed fluid: serum Analythical methodology: radioimmunoassay Vit. D mean (SD) (nmol/L): Summer 65,5 (SD 25,2); Winter 39.2 (SD 14.4) Vit. D status: NA Dietary vit. D intake: categorized as none, 0–200 IU/day, 201–750 IU/day.	Evaluation of depressive symptoms: Hospital Anxiety and Depression Scale (HADS-D) Depressive symptoms severity: NA	Sun exposure: Not Measured Fatigue Diagnosis: The Fatigue Severity Scale (FSS) Degree of disability: Expanded Disability Status Score (EDSS) Cognitive Performance: 3-s Paced Auditory Serial Addition Test (PASAT-3)	Aim: Examining the associations between personal sun exposure and serum 25- hydroxyvitamin D (25(OH)D), and depression, anxiety, fatigue and cognition. Results: 25(OH)D levels were not associated with depression scores. Higher levels of reported personal sun exposure in the current season were associated with lower depression score. Effect size: correlation, β = −0.05 (−0.12–0.02) (*p*-value = 0.186) Controlled cofounders: sex, age, initial EDSS score, initial disease duration, immunomodulatory therapies, BMI and season Quality assessment (NOS): Selection (max 4): 2 - Representativeness: 1 - Selection of the non exposed: 1 - Ascertainment of exposure: 0 - Outcome not present at start: 0 Comparability (max 2): 2 Outcome (max 3): 2 - Assesment: 0 - Follow-up lenght: 1 - Adequacy of follow up: 1 Total score (max 9): 6
Rolf et al., 2017 (The Netherlands) D: Randomized Placebo controlled trial (RCT) RT: FU: 48 weeks Participants: 40 (20 with vitD; 20 with Placebo) Female/Male: 26/14 (VitD 12/8; Placebo 14/6) Mean Age (SD): VitD 37.6 (9.6); placebo 38.5 (7.8) Diagnosis MS: McDonald’s criteria Clinical Status: Being relapse-free for 30 days prior to participation, having had their first clinical event in the previous 5 years, not active in the 30 days prior to inclusion, RR MS, treated with interferon-β1α.	Analyzed fluid: serum Analythical methodology: radioimmunoassay Vit. D median (Q1–Q3) (nmol/L): baseline control 53 (Q1-Q3 43–63), baseline case 58 (Q1–Q3 38–82); endpoint: control 61 (44–84), case 226 (159–250) Vit. D status: NA Dietary vit. D intake: NA NB: D3 supplementation in the vit D group cholecalciferol dosed at 7000 IU/day in the first 4 week, followed by 14,000 IU/day up to endpoint.	Evaluation of depressive symptoms: Hospital Anxiety and Depression Scale (HADS-D) Depressive symptoms severity: baseline control 3.0 (Q1–Q3 2.0–7.0), baseline case 4.0 (Q1–Q3 2.0–5.0)	Sun exposure: participants quantified how much time they spent in the sun during weekends and holidays in the current and preceding 3-month intervals using validated questions Fatigue Diagnosis: The Fatigue Severity Scale (FSS) Inflammation Status: assessment of TNFα and IL-10 concentrations	Aim: Exploring the effect of high dose vitamin D3 supplementation on depressive symptoms in MS. Fatigue was assessed as a potential confounder. Results: High dose vitamin D supplementation does not decrease depression and fatigue scores. Effect size: non significant, effect size not reported Controlled cofounders: HADS-D at baseline and FSS at T1 Quality assessment (Cochrane Risk of Bias 2.0): Randomization process: Some concerns Deviations from intended interventions: High risk Mising outcome data: Some concerns Measurement of the outcome: High risk Selection of the reported result: Some concerns Overall Bias: High risk

*: poster; IQR: interquartile range; SD: standard deviation; D: study design; RT: recruitment timing; FU: follow-up; NR: not relevant; NA: not available; RR MS: Relapsing–Remitting Multiple Sclerosis.
